# COVID-19 and Its Lockdown in Belgium: How Limited Access to Environmental Satisfaction Impacts Emotions?

**DOI:** 10.5334/pb.1082

**Published:** 2022-01-20

**Authors:** Aurélie Wagener, Céline Stassart, Anne-Marie Etienne

**Affiliations:** 1 Research Unit for a life-Course perspective on Health and Education (RUCHE), Health Psychology, Department of Psychology, Faculty of Psychology and Science of Education, University de Liege, Rue de l’Aunaie, 30, 4000, Liege, BE

**Keywords:** COVID-19, Lockdown, Environmental Satisfaction, Emotions

## Abstract

The COVID-19 pandemic has been shown to induce several psychological consequences (e.g., increases in anxiety and stress). Accordingly, it seems relevant to investigate how individuals cope with their emotions. Indeed, when facing negative emotions, individuals need to rely on adaptive emotion regulation strategies to alleviate their negative impacts (e.g., decrease in quality of life, increase in sleep disturbances). Moreover, lockdown’s measures led to a restriction of the access to activities which, in turn, might have decreased the “environmental satisfaction”. Then, this research investigates the pandemic’s psychological impacts on emotions and regulation strategies, intolerance of uncertainty, and environmental satisfaction. Our approach’s originality relies on comparing one’s actual psychological functioning (i.e., since the pandemic) to one’s general psychological functioning (i.e., before the pandemic). This study also assesses the relationships between both negative and positive emotions and (1) emotion regulation strategies, (2) intolerance of uncertainty and, (3) environmental satisfaction since the pandemic and its lockdown. The total sample comprised 948 adults from the general population. Results show that, since the pandemic, individuals experience higher levels of negative emotions, lower levels of positive emotions and environmental satisfaction. They also tend to worry less and to resort to acceptance more often. Also, environmental satisfaction is the most important predictor of both negative emotions and positive ones. Overall, findings confirm previous ones and seem to indicate that environmental satisfaction should be addressed more thoroughly.

## Introduction

While the COVID-19 pandemic and its consequences are an integral part of our everyday life, let us remember how the World’s functioning has radically changed over the past year. Wuhan, 31^st^ of December 2019: a cluster of cases of pneumonia is identified. Wuhan, 7^th^ of January 2020: the coronavirus “SARS-CoV-2” (COVID-19) is identified as the cause of pneumonia. Geneva, 30^th^ of January 2020: the World Health Organization (WHO) states that the outbreak of COVID-19 constitutes a Public Health Emergency of International Concern (WHO, 2020a). Brussels, 4^th^ of February 2020: the first case of COVID-19 in Belgium is diagnosed. Geneva, 11^th^ of March 2020: the outbreak of COVID-19 is considered as a World Pandemic (WHO, 2020b). Brussels, 18^th^ of March 2020: a generalized lockdown is applied. Indeed, facing the rapid and worrying spreading of the COVID-19, politics had to make a decision to contain the pandemic’s evolution. In Belgium, a generalized and long-term lockdown has been applied to limit physical contacts which are at the origin of the contaminations. Concretely, social activities were forbidden. In addition, schools, recreational centers (e.g., sport clubs, cinemas) and non-essential shops (e.g., bookstores, hairdressers) have been closed. Also, when the professional function was suitable for, homeworking has been applied while when the professional function was not eligible for homeworking, individuals were technically unemployed. In the case of meeting with other people (e.g., in the supermarket), protective behaviors such as wearing a facial mask, use of sanitizers and handwashing had to be applied. Even though those decisions led to beneficial effects on the dissemination of the virus, they nonetheless had significant psychological consequences (Restubog, Ocampo, & Wang, 2020).

Since the onset of the pandemic, an important number of studies on its psychological impacts has been published. A majority of those assessed its emotional impacts and showed that, in general, individuals experience a decrease in their quality of life (Bao, Sun, Meng, Shi, & Lu, 2020; Khodami, Seif, Koochakzadeh, Fathi, & Kaur, 2021; Porcelli, 2020). To summarize main results, Rajkumar (2020) performed a meta-analysis and showed that mental health during the current pandemic is characterized by increases in anxiety, fear, stress, depression, and sleep disturbances. Pollard, Tucker, and Green (2020) also evidenced increases in alcohol consumption while Nutley et al. (2021) highlighted increases in disordered eating behaviors. While Taylor et al. (2020) advised that more than 25% of the general population are prone to experience moderate to severe anxiety and stress levels, fear appears to be the most reported emotion. Indeed, individuals experience fear of getting ill or dying, of the professional consequences (e.g., job insecurity, decrease of their income), and of the social ones (e.g., loneliness, loss, or deterioration of relationships) (Porcelli, 2020; Taylor et al., 2020; Van Bavel et al., 2020). Anxiety, fear and stress could be due to the uncertainty inherent to any pandemic situation. Uncertainty can be defined as the “*presence of vague, complex or unpredictable stimuli or conditions and insufficient or inconsistent information to deal with them*” (Del Valle et al., 2020). Regarding the COVID-19 pandemic, uncertainty concerns the unpredictability of the future (i.e., the inability to predict the course of the disease and its related events) as much as the limited ability to exert control over the situation. Facing situations of uncertainty, some individuals might experience “*uncertainty distress*”, defined by Freeston, Tiplady, Mawn, Bottesi, and Thwaites (2020) as “*the subjective negative emotions experienced in response to the as yet unknown aspects of a given situation”*. This distress due to uncertainty could, in turn, render individuals intolerant to uncertainty. Del Valle et al. (2020) demonstrated that – in the context of the pandemic – intolerance of uncertainty is a significant predictor of both anxiety and depression.

In regards to the previous paragraph, the negative emotional impacts of COVID-19 seem to be well-established. Consequently, it seems relevant to investigate how individuals cope with these emotions. Indeed, when facing negative emotions, individuals need – among other things – to rely on adaptive and effective emotion regulation strategies to alleviate their negative impacts (e.g., decrease in quality of life, increase in sleep disturbances) (Restubog et al., 2020). In 2007, Garnefski and Kraaij defined cognitive emotion regulation as conscious strategies used to cope with emotionally arousing events and/or information. The use of adaptive emotion regulation strategies (e.g., acceptance, positive reappraisal) has been linked to higher levels of quality of life and well-being, while the use of non-adaptive ones (e.g., worry/rumination, self-blame) has been related to lower levels of quality of life and well-being (Balzarotti, Biassoni, Villani, & Velotti, 2016; Garnefski & Kraaij, 2007). While some studies confirmed the relationship between emotion regulation strategies and quality of life during the COVID-19 pandemic (e.g., Dubey, Podder, & Pandey, 2020; Khodami et al., 2021), modifications in the use of emotion regulation strategies since the pandemic have not yet been investigated. Consequently, it seems of interest to compare the use of emotion regulation strategies in general to the use of these strategies in the specific pandemic context to investigate their potential evolution when facing such adverse situations.

Beyond the above-mentioned emotional impacts of the COVID-19, it seems relevant to focus on the specific impact of lockdown’s measures. In Belgium, these measures led to the closing of almost all establishments (e.g., non-essential shops such as bookshops or garden centers, cinemas, sports halls) and the cessation of almost all activities (e.g., hobbies such as cultural activities or artistic ones). Further, social contacts were strictly limited. Overall, those protective measures led to an extreme restriction of the access and the engagement in activities (Cruyt et al., 2021). In turn, this might have impeded the sense of “*environmental satisfaction*,” which is defined as “*one’s perception of the positive or negative value of environmental experiences and activities available in its environment*” (Wagener & Blairy, 2015). Environmental satisfaction can be experienced in several life domains (e.g., relationships, work, education, hobbies). Nonetheless, it is noteworthy that its level depends on personal interests and values and is likely to vary across individuals. For instance, some individuals may enjoy gardening and taking walks, while others may enjoy biking and taking photographs. The impact of a limited access to pleasure or reinforcement (i.e., low environmental satisfaction) is thoroughly assessed since the seventies (e.g., Lewinsohn, 1975; Lewinsohn & Amenson, 1978; Lewinsohn & Graf, 1973). Overall, previous research reported that the engagement in pleasant or mandatory activities is positively related to well-being (Carvalho, Trent, & Hopko, 2011; Mazzucchelli, Kane, & Rees, 2010), while the lack of engagement in such activities is positively linked to symptoms of depression (Wagener, Baeyens, & Blairy, 2016). By the way, these principles consist in the core components of a well-established treatment of depression, namely behavioral activation, which aims at (re)engaging one’s in pleasant and/or mandatory activities (Hopko, Lejuez, Ruggiero, & Eifert, 2003; Kanter et al., 2010). In accordance with the previous, it seems reasonable to hypothesize that the COVID-19 lockdown led to a decrease in environmental satisfaction, which in turn might have elicited negative emotions or even depressive symptoms. Until now, while perceived by a majority of persons, the relationship between environmental satisfaction and emotions in the pandemic’s specific context has not been assessed. In April 2021, as the pandemic is still ongoing and that lockdown measures are maintained, it appears sensitive. From a political perspective, if environmental satisfaction proves to be an important predictor of emotions, this might give insight into the future health recommendations to handle the spread of the COVID-19. From a clinical perspective, this might help enlighten the need to return to behavioral activation roots as well as the need to tailor this intervention to be more appropriate to the actual context.

This study’s aim was twofold. First, we aimed at investigating the psychological impacts of the COVID-19 on emotions and regulation strategies, intolerance of uncertainty, and environmental satisfaction. Then, our first outcome is to appreciate changes in the aforementioned variables since the pandemic. Our approach’s originality relies on comparing one’s actual psychological functioning (i.e., since the pandemic) to one’s general psychological functioning (i.e., before the pandemic). We hypothesized that we would replicate previous results. More precisely, we expected to show an increase in negative emotions and intolerance of uncertainty along with a decrease in positive emotions and environmental satisfaction during the lockdown when compared to one’s general state. In regards to emotion regulation strategies, several hypotheses are possible as the examination of this variable is quite exploratory. If a disturbance in emotional regulation is observed, we expect individuals to resort more often to non-adaptive emotion regulation strategies and less often to adaptive ones since the pandemic. Further, putting an emphasis on environmental satisfaction, we aimed at offering a descriptive and exploratory overview of the actual activities in which individuals were able to engage themselves in during the lockdown. Second, we aimed at investigating the relationships between both negative and positive emotions and (1) emotion regulation strategies, (2) intolerance of uncertainty and, (3) environmental satisfaction since the pandemic and its lockdown. More precisely, the extent to which emotion regulation strategies, intolerance of uncertainty, and environmental satisfaction consisted in predictors of negative emotions and positive ones was assessed. In other words, our second outcome is to highlight the variables which predict the appearance of negative emotions and/or positive ones. We hypothesized that negative emotions would be (1) positively predicted by worry and intolerance of uncertainty and (2) negatively predicted by positive reappraisal, acceptance, and environmental satisfaction. The inverse hypotheses were stated for positive emotions.

## Methods

### Participants and Procedure

Power analyses were performed a priori using G*Power 3.1. (Faul, Erdfelder, Buchner, & Lang, 2009). Applied to our t test for paired samples (t tests; means: difference between two dependent means – matched pairs), the power calculation (power of 0.95, α-error of 0.05, an estimated effect size of 0.5) indicated that a sample of 45 participants was requested to detect effects. Applied to our multiple linear regression (t tests; linear multiple regression: fixed model, single regression coefficient), the power calculation (power of 0.95, α-error of 0.05, an estimated effect size of 0.15, five predictors) indicated that a sample of 74 participants was requested to detect effects.

The current sample comprised 948 adults (681 women) from the general population with an average age of 40.45 (range = 18–79, SD = 13.74). In Belgium, a first lockdown was applied from March to June 2020. From May to June 2020, adults from the general population were invited to take part in the study through advertisements on social networks and university websites’ announcements. The study’s aim was described, and a link to an online survey was provided. The survey included a socio-demographic questionnaire as well as other measures, which are described in the next section. The administration of these scales was part of a more comprehensive evaluation process that included other self-reported measures such as comparative optimism and application of sanitary measures. As the current paper focuses on emotional aspects, the other self-reported measures were not involved since they rather address health beliefs and behaviors than emotions. The local ethics committee of our college of psychology approved the protocol (approval number: 1920–97), and all participants provided online informed consent.

### Measures

#### Demographic information

Participants had to indicate their age, gender, and country of residence. They were also asked to determine their relationship status and to indicate if they have children. Regarding work situation, they had to indicate if their working modalities were identical to those before the COVID-19 pandemic or have been modified (i.e., homeworking, technical lay-off).

#### Activities information

Participants had to report if they engaged themselves in novel activities in different life areas, namely: physical activities (e.g., go cycling or for a walk); cooking; gardening; artistic activities (e.g., painting, sculpting); well-being activities (e.g., yoga, meditation) or COVID-19’s helping activities (e.g., sewing facial masks, neighbor errands). These life areas were selected based on the Brief Behavioral Activation Treatment for Depression – Revised version (Lejuez, Hopko, Acierno, Daughters, & Pagoto, 2011). In this treatment protocol, the authors mention ten life areas which cover a vast majority of activities: family relationships, social relationships, romantic relationships, education/training, employment/career, hobbies/recreation, volunteer work/charity/political activities, physical and psychological health issues, spirituality, daily responsibilities. Due to lockdown, current and novel activities in some of these life areas were forbidden (e.g., social relationships, romantic relationships). Then, we assessed the remaining life areas in which individuals were able to engage in novel activities (i.e., physical and psychological health issues, hobbies/recreation, volunteer work/charity/political activities, daily responsibilities).

#### Basic Emotions Scale

The Basic Emotions Scale (Philippot, 2011; Power, 2006) assesses the five clusters of basic emotions (i.e., happiness, anger, anxiety, sadness, disgust) through 20 different emotional terms (e.g., happy, frustrated, anxious, depressed, blameworthy). Participants were asked to assess the extent to which the 20 emotions were experienced *during the last week* and *in general*, to allow emotions’ comparison during the pandemic’s lockdown and before the pandemic. Each emotion is rated on a 7-point Likert scale (1 = Not at all, 7 = All the time). A score can be calculated for each cluster of emotions by averaging scores of its component emotions. Internal consistencies were satisfactory since Cronbach alphas ranged from 0.79 to 0.84 (Power, 2006).

#### Emotion Regulation Strategies, Intolerance of Uncertainty and Environmental Satisfaction

To assess these three variables, we selected items from four different scales to limit the length of the protocol. The selection of the items was based on their factor loadings on the reference scale: on each scale, the two items with the higher factor loadings were selected. For all variables, individuals were asked to assess the items *since the onset of the pandemic* and *in general*. Each item is rated on a 5-point Likert Scale (1 = Totally disagree, 5 = Totally agree). For each of the aforementioned variables, a mean of the two items was calculated to represent the current score, on the one hand, and the general score, on the other hand.

Regarding emotion regulation strategies, acceptance and positive reappraisal were assessed through items retrieved from the Cognitive Emotion Regulation Questionnaire (e.g., “*I think that I have to accept the situation*”, “*I think I can learn something from the situation*”) (Garnefski, Kraaij, & Spinhoven, 2001; Jermann, Van der Linden, d’Acremont, & Zermatten, 2006). Worry was assessed through items retrieved from the Penn State Worry Questionnaire (e.g., “*I know I should not worry about things, but I just cannot help it*”) (Gosselin, Dugas, Ladouceur, & Freeston, 2001). Factor loadings ranged between 0.77 and 0.79.

Regarding intolerance of uncertainty, items were retrieved from the Intolerance of Uncertainty Scale (e.g., “*I should be able to organize everything in advance*”) (Buhr & Dugas, 2002; Freeston, Rhéaume, Letarte, Dugas, & Ladouceur, 1994). Factor loadings ranged between 0.58 and 0.74.

Regarding environmental satisfaction, items were retrieved from the Environmental Reward Observation Scale (e.g. “*My life is boring*”, “*It is easy for me to find enjoyment in my life*”) (Armento & Hopko, 2007; Wagener & Blairy, 2015). Factor loadings ranged between 0.79 and 0.83.

### Statistical Analyses

Statistical analyses were performed as follows. First, we compared the psychological state during the last week to the general psychological state using t tests for paired samples. Second, we investigated the extent to which individuals engaged themselves in novel activities through descriptive statistics. Third, we assessed the predictive value of five independent variables (i.e., environmental satisfaction, intolerance of uncertainty, worry, positive reappraisal, acceptance) on negative and on positive emotions by running multiple regression analyses.

Alpha was set at 0.05. However, given the number of statistical analyses and the need to balance the amount of type 1 and type 2 errors, we calculated adjusted p values with the false discovery rate method for multiple testing that is the Benjamini-Hochtberg Index (Benjamini & Yekuteli, 2001). Briefly, the false discovery rate controls the expected proportion of falsely rejected null hypotheses. This method has been shown to be much more powerful than methods that control the familywise error rate (e.g., Bonferroni) (Benjamini & Hochtberg, 1995; Benjamini & Yekuteli, 2001). All analyses were performed with SPSS v.26.

### Results

#### Demographic Data

Demographic characteristics are displayed in ***Table 1***.

**Table 1:** Sociodemographic characteristics.


		n	%

Gender	Male	267	28.2

	Female	681	71.8

Marital status	Single	230	24.3

	Single, with children	54	5.7

	In a relationship	325	34.3

	In a relationship, with children	339	35.8

Working status	As usual	206	21.7

	Homeworking	452	47.7

	Technichal lay-off	33	3.5

	Unemployment	124	13.1

	Student	132	13.9

	Other (i.e., retired, on sick leave)	1	0.1


#### Psychological State: Last Week vs In General

The ***Table 2*** presents results of t tests for paired samples which were performed to compare the current psychological state to the general one (Benjamini-Hochtberg Index = 0.02).

**Table 2:** Comparison of the psychological state between last week and in general.


		RANGE	LAST WEEK	GENERAL STATE	*t (df)*	*p*

			*M (SD)*	*M (SD)*		

Emotions	Joy	1–7	3.66 (1.37)	4.21 (1.34)	–13.93 (947)	<0.0001

	Anger	1–7	3.40 (1.36)	2.24 (1.05)	30.44 (947)	<0.0001

	Fear	1–7	3.60 (1.50)	3.13 (1.40)	12.71 (947)	<0.0001

	Sadness	1–7	2.49 (1.34)	1.86 (1.00)	18.46 (947)	<0.0001

	Disgust	1–7	2.47 (1.71)	1.80 (1.28)	13.43 (947)	<0.0001

Emotion regulation strategies	Worry	1–5	2.94 (1.01)	3.09 (1.12)	–6.13 (947)	<0.0001

	Positive reappraisal	1–5	4.01 (0.93)	4.06 (0.81)	–1.52 (947)	0.13

	Acceptance	1–5	3.81 (1.01)	3.68 (0.96)	4.51 (947)	<0.0001

Intolerance of uncertainty		1–5	3.63 (1.20)	3.59 (1.15)	1.21 (947)	0.23

Environmental satisfaction		1–5	3.66 (1.00)	4.02 (0.91)	–13.09 (947)	<0.0001


As showed in ***Table 2***, all emotions were significantly different between last week and the general state. More precisely, the level of joy was lower during the last week than in general, while all negative emotions were higher. Regarding emotion regulation strategies, differences were only observed on worry and acceptance: the level of worry was lower, and the level of acceptance was higher. No significant difference was shown on the intolerance of uncertainty. Environmental satisfaction was significantly lower during the last week when compared to the general level.

#### Engagement in Activities: What Is New in What People Do?

Two hundred fifty seven participants indicated that they did not engage themselves in new activities since the lockdown (27.10% of the total sample). In the remaining 691 participants, 274 (39.65%) started cooking; 268 (38.78%) started physical activities (e.g., go cycling or for a walk); 250 (36.18%) started gardening; 142 (20.55%) engaged in COVID-19’s helping activities (e.g., sewing facial masks, neighbor errands); 124 (17.95%) started artistic activities (e.g., painting, sculpting), and 120 (17.37%) started well-being activities (e.g., yoga, meditation).

#### Assessment of the Predictive Values of our Independent Variables on Negative Emotions and Positive Emotions

The regression analyses assessed the predictive values of emotion regulation strategies (i.e., worry, positive reappraisal, acceptance), intolerance of uncertainty, and environmental satisfaction on emotions by running multiple regression analyses (Benjamini-Hochtberg Index = 0.02) (***Tables 3*** and ***4***). No sign of multicollinearity was evidenced for any of the assessed variables (VIF < 10), worry (VIF = 1.63), acceptance (VIF = 1.05), positive reappraisal (VIF = 1.14), intolerance of uncertainty (VIF = 1.61), environmental satisfaction (VIF = 1.31).

**Table 3:** Results of the multiple regression analyses on negative emotions.


DEPENDENT VARIABLE	INDEPENDENT VARIABLES	R^2^	ADJUSTED R^2^	STANDARD ERROR OF THE ESTIMATE	F	df	p	β	t	p	COLINEARITY STATISTICS	

											TOLERANCE	VIF

Negative emotions		0.24	0.23	0.76	58.50	5	<0.0001					

	Worry							0.19	5.15	<0.0001	0.61	1.63

	Positive reappraisal							0.06	2.10	0.04	0.88	1.14

	Acceptance							–0.06	–2.18	0.03	0.95	1.05

	Intolerance of uncertainty							0.17	4.83	<0.0001	0.62	1.61

	Environmental satisfaction							–0.26	–7.91	<0.0001	0.77	1.31


**Table 4:** Results of the multiple regression analyses on positive emotions.


DEPENDENT VARIABLE	Independent variables	R^2^	ADJUSTED R^2^	STANDARD ERROR OF THE ESTIMATE	F	df	p	β	t	p	COLINEARITY STATISTICS	

											TOLERANCE	VIF

Positive emotions		0.15	0.14	1.24	32.91	5	0.0001					

	Worry							–0.00	–0.05	0.96	0.61	1.63

	Positive reappraisal							0.13	3.90	<0.0001	0.88	1.14

	Acceptance							–0.02	–0.52	0.60	0.95	1.05

	Intolerance of uncertainty							0.02	0.53	0.60	0.62	1.61

	Environmental satisfaction							0.34	9.75	<0.0001	0.77	1.31


Negative emotions were positively predicted by intolerance of uncertainty and worry (β = 0.17, *p* < 0.0001, and β = 0.19, *p* < 0.0001, respectively) and negatively predicted by environmental satisfaction (β = –0.26, *p* < 0.0001). Acceptance and positive reappraisal were not significant predictors of negative emotions (β = –0.06, *p* = 0.03, and β = 0.06, *p* = 0.04, respectively).

Positive emotions were positively predicted by positive reappraisal and environmental satisfaction (β = 0.13, *p* < 0.0001, and β = 0.34, *p* < 0.0001, respectively). Acceptance, intolerance of uncertainty and worry were not significant predictors of positive emotions (β = –0.02, *p* = 0.60; β = 0.02, *p* = 0.60; β = –0.002, *p* = 0.96, respectively).

## Discussion

At a period characterized by the threat of a new wave of coronavirus, it appears necessary to understand pandemic’s psychological consequences as much as their underlying phenomena. This approach to the current context might help to accurately accompany the whole population, from a psychological perspective (e.g., primary prevention, adjustments of usual clinical tools) as well as a political one (e.g., defining sanitary measures to contain the pandemic based on psychological knowledges). This seems all the more important since other epidemics have already marked history and because it is quite likely that the World will again be confronted with other pandemics. It is with this in mind that we conducted a study focusing on psychological impacts of the COVID-19 pandemic.

The first aim of the present study was to investigate the psychological impacts of the COVID-19 on emotions and their regulation strategies (i.e., worry, positive reappraisal, acceptance), intolerance of uncertainty and environmental satisfaction. These impacts have been assessed through the comparison of one’s current psychological functioning to its general psychological functioning. In regards to previous research on emotions, our study replicated the highlighted results (Rajkumar, 2020). More concretely, participants reported higher levels of negative emotions during the last week than in general while they also reported lower levels of positive emotions during the last week than in general. Concerning emotion regulation strategies, results were more mixed. A decrease in worry was observed which might appear unexpected in the pandemic’s context. Nonetheless, different explanations are suggested. Firstly, the perceived threat might have been alleviated since physical contacts – which are the source of contaminations – were restricted. Further, the perceived control over the situation might have been enhanced due to the lockdown. In turn, this might have appease the extent to which one worries. Secondly, one’s overall mental load might have been calmed down due to the lockdown. Indeed, daily organization has been modified and a series of activities has been retrieved which might, in turn, have eased one’s agenda and then, its tendency to worry. Further, an increase in acceptance was showed which seems sensitive when referred to its definition as “*thoughts of accepting what you have experienced and resigning yourself to what has happened*” (Garnefski & Kraaij, 2007). When the study was conducted (May and June 2020), we were still in the first months of the pandemic which was characterized by important sanitary measures to contain the spread of the coronavirus. The whole population did not have other choice than applying political recommendations. Consequently, resorting to acceptance appears to be quite adaptive at that phase of the pandemic. Nonetheless, it is noteworthy that accepting the situation is to be distinguished from the negative emotions that are experienced. Accepting the situation does not prevent from the appearance of negative emotions but might help to alleviate those emotions. No significant difference was underlined on positive reappraisal and on intolerance of uncertainty. Regarding positive reappraisal, it seems relevant to remind its definition as “*thoughts of creating a positive meaning to the event in terms of personal growth*” (Garnefski & Kraaij, 2007). When they participated in our study, individuals were still in the adaptation phase to the pandemic situation since it was ongoing for the last two months. In other words, they were still adjusting to challenges such as juggling with homeworking and school at home. Consequently, they were certainly not able and available to invest themselves in deep personal growth which is aimed when one recourses to positive reappraisal. This seems in accordance with the literature on identity which indicates that personal growth requires efforts to integrate new and different roles or experiences in a whole coherent identity (Diehl & Hay, 2011). Regarding intolerance of uncertainty, it seems relevant to dive back in Freeston et al. (2020) who distinguished the *trait* of being intolerant to uncertainty to the *emotional state* of being distressed due to uncertainty. The current study has been conducted during the second month of the pandemic. It seems quite possible that psychological characteristics which define one’s functioning (e.g., intolerance of uncertainty) have not been modified in such a short period of time while the emotional state has been proven to evolve (Rajkumar, 2020). As expected, a decrease in environmental satisfaction was observed. Even though this result might appear quite logical, it remains important to rely on empirical evidences confirming popular perceptions. In order to encounter environmental satisfaction again, even though more than 25% of the participants did not report any new activity, it seems that a majority of our sample engaged in novel activities. The most frequent activities were cooking, physical activities as well as gardening. At this point, it is important to report a limitation of our study: individuals had to select activities in a predetermined list. They were not able to select other areas (e.g., home improvement) or indicate other specific activities (e.g., online games, online aperitifs). Consequently, the description of the type of activities might lack accuracy. Further, participants were asked to indicate if they engaged themselves in *novel* activities. Actually, it might have been more accurate to also ask if they engaged themselves *more frequently* in *usual* activities. Another limitation should be mentioned in regards to the interpretation of the results on activities. The data do not allow to appreciate if the engagement in activities is actually positive for the individuals. For instance, an important portion of the sample reported cooking as a novel activity. This might be pleasant and satisfactory but can also be perceived as boring. These issues should be addressed in future studies.

The second aim of the present study was to assess the extent to which (1) emotion regulation strategies, (2) intolerance of uncertainty and, (3) environmental satisfaction are significant predictors of both negative and positive emotions. While these relationships have already been investigated in previous studies (Balzarotti et al., 2016; Freeston et al., 2020; Wagener & Blairy, 2015), their assessment in the specific pandemic’s context remains relevant. Indeed, these relationships might appear modified which, in turn, could implicate adjustments in clinical practice. In the current study, the most important predictor of both negative and positive emotions was environmental satisfaction. In other words, the more you perceive environmental satisfaction, the less negative and the more positive your emotions will be. Overall, although stemming from a specific context, these results confirmed previous findings (Carvalho et al., 2011; Mazzucchelli et al., 2010; Wagener et al., 2016). Environmental satisfaction is reached through the contact with sources of pleasure and/or reinforcement (Wagener & Blairy, 2015). As mentioned above, it appears impeded since the onset of the COVID-19 pandemic. In light of its decrease, on the one hand, and its impact on experienced emotions, on the other hand, environmental satisfaction should be one of the therapeutic targets as discussed in the clinical perspectives below. In regards to emotion regulation strategies, even though all the three assessed strategies did not prove to be significant predictors of emotions, our results confirmed their link with negative emotions as those of Dubey et al. (2020). More precisely, worry positively predicted negative emotions which is a classic observation (Szabó, 2011). Positive reappraisal positively predicted positive emotions which is consistent with the scientific literature (Nowlan, Wuthrich, & Rapee, 2016). Finally, negative emotions were positively predicted by intolerance of uncertainty which confirms previous findings (Del Valle et al., 2020). Overall, these results were in line with the framework supported by Freeston et al. (2020) stating that distress uncertainty is related to the unpredictability of the future (i.e., the inability to predict the course of the disease and its related events) and the limited ability to exert control over the situation such as experienced with the COVID-19 pandemic.

Briefly, our results confirmed previous findings on the consequences of the pandemic (e.g., Del Valle et al., 2020; Dubey et al., 2020; Rajkumar, 2020). This seems particularly noteworthy considering that our discipline undergoes a “replication crisis” (Wiggins & Chrisopherson, 2019). Further, our results highlighted the presence of well-known psychological phenomena in the specific context of the pandemic (i.e., links between environmental satisfaction, worry, positive reappraisal, intolerance of uncertainty, and emotions, respectively). This has the advantage of enabling clinical recommendations based on empirically grounded principles and guidelines, as discussed in the next section. Also, this might be comforting for clinicians who are able to rely on their usual practice even though their patients and themselves face a crisis.

### Perspectives

This study offers several clinical and experimental perspectives. From a clinical perspective, we confirmed the experience of negative emotions at the onset of the pandemic. While unpleasant, those emotions have been proven to also incite individuals to apply protective behaviors as recommended (e.g., physical distance, handwashing) (Bigot, Banse, Cordonnier, & Luminet, 2021; Harper, Satchell, Fido, & Latzman, 2020). Nonetheless, alleviating negative emotions seems essential to prevent the development of subsequent mental health issues. Indeed, long lasting negative emotions consist in one of the most important risk factors to mental illness (Piqueras, Ramos, Martínez González, & Oblitas Guadalupe, 2009). This study also evidenced the presence of a series of well-known phenomena (e.g., the relationship between emotions and environmental satisfaction), even in the specific context of the COVID-19 pandemic. However, despite the fact that these phenomena are usual, their prevalence might be more important during the pandemic. In general, our results supported the use of our usual clinical tools if possible but also emphasized the need to adapt these interventions to the current sanitary recommendations and to the widening of psychological help requests. Then, the development of first line psychological interventions seems of interest. Indeed, offering an intervention soon enough might help in reducing mental health burden in the general population. This might be achieved through self-help brochures or websites which efficiency has already been evidenced for different psychological issues (e.g., Cuijpers, Donker, van Straten, & Andersson, 2010; Parks & Szanto, 2013). According to our results, these self-help interventions should offer therapeutic education on worry, positive reappraisal, intolerance of uncertainty and, environmental satisfaction.

Beyond delivering pieces of information, our results also indicated the relevance of reducing the tendency to worry. This might be achieved through the development of more concrete ways of thinking and the disengagement from emotional responses (McCarrick, Prestwich, Prudenzi, & O’Connor, 2021). Further, an intervention specifically dedicated to the development of positive reappraisal seems of interest since it is positively linked to positive emotions. This seems all the most relevant as positive reappraisal consists in an emotion regulation strategy that allows individuals to adjust adequately to stressful events. In order to enhance the use of this strategy, Garland, Gaylor, and Park (2009) suggested the recourse to mindfulness. Actually, Behan (2020) also highlighted the benefits of meditation and mindfulnesss practice during crisis periods such as the COVID-19 pandemic. In our digital period, it is noteworthy that smartphones applications already offer mindfulness programs, freely for some of them.

In regards to the decrease in environmental satisfaction, the recommended intervention should be behavioral activation. As explained above, behavioral activation aims at (re)engaging one’s in pleasant and/or mandatory activities (Hopko et al., 2003; Kanter et al., 2010). While empirically grounded, this rationale seems complicated to apply in the context of a pandemic since it implies the actual access to several activities, a certain amount of them needing infrastructures outside the home and requiring social presence. This underlines the need to creatively apply behavioral activation’s rationale and principles while applying sanitary recommendations (e.g., physical distance, limitation of social contacts).

In regards to experimental perspectives, the distinction of the “current psychological functioning” versus the “general psychological functioning” should be more thoroughly invested. Actually, the lack of differences between one’s actual state and its general one might be explained by the assessment of trait (e.g., intolerance of uncertainty) rather than state (e.g., uncertainty distress) variables. Future studies should differentiate more accurately these two notions. Future research could find interest in the use of the “COVID Stress Scales” developed by Taylor et al. (2020) in the specific context of the pandemic. This scale might offer a more accurate evaluation since it assesses five dimensions related to one’s experience of the pandemic (i.e., danger and contamination, socioeconomic consequences, xenophobia, traumatic stress symptoms, compulsive checking). Since the pandemic continues, longitudinal studies might help understanding more accurately the adaptation to the pandemic. For instance, negative emotions have been proven to incite the application of protective behaviors at the onset of the pandemic. A year later, we may wonder whether these conclusions are still valid, especially since compliance with barrier gestures seems to decrease with the length of the pandemic (e.g., Ning et al., 2020). Accordingly, mental health issues – on which an important number of studies focused since the onset the pandemic – should be at the heart of the actual research as much as the pandemic is still ongoing. Indeed, deep detailed assessment of mental health issues, including diagnosis indicators (e.g., generalized anxiety disorder, major depressive disorder), might allow more accurate primary prevention. Finally, until now, studies mainly focused on the negative impact of the pandemic. Nonetheless, it remains possible that positive effects have appeared since the pandemic. For example, Nelson (2020) and Sandin, Valiente, García-Escalera, Campgne, & Chorot (2020) mentioned the reassessment of priorities and values, beyond positive effects on the environment.

## Limitations

Our results should be interpreted in the light of four limitations. First, participants were asked to assess their general psychological state while they were currently adapting to the pandemic and its consequences. Their responses might suffer from a mood congruence bias. Second, the current research assessed the relationships between several predictors and positive or negative emotions but we are not able to determine any causal effect. Third, our sample is mainly composed of women. Then, our results might be quite different in a more balanced sample. Fourth, information on children lacks precision since participants did not have to indicate the number of children, their age, if they were still living in the same house, if they had to accompany learning activities at home. Yet, these different elements have been proven to influence one’s well-being since the pandemic (e.g., Stassart, Wagener, & Etienne, 2021).

## Conclusion

The COVID-19 pandemic has several psychological consequences. Overall, individuals experience higher levels of negative emotions along with lower levels of positive ones. They also tend to worry less and accept more. Further, environmental satisfaction appears weakened. In regards to this variable, it appears that environmental satisfaction is the most important predictor of both negative emotions and positive ones. Therefore, it consists in a variable of interest for clinical practice and should certainly be considered in the future recommendations to handle the pandemic.
